# Broadband giant nonlinear response using electrically tunable polaritonic metasurfaces

**DOI:** 10.1515/nanoph-2023-0682

**Published:** 2024-01-09

**Authors:** Jaeyeon Yu, Seongjin Park, Inyong Hwang, Gerhard Boehm, Mikhail A. Belkin, Jongwon Lee

**Affiliations:** Department of Electrical Engineering, Ulsan National Institute of Science and Technology, Ulsan, 44919, Republic of Korea; Walter Schottky Institute, Technical University of Munich, Am Coulombwall 4, 85748 Garching, Germany

**Keywords:** metasurface, nonlinear optics, second harmonic generation, reconfigurable, broadband, intersubband transitions

## Abstract

Intersubband transitions in semiconductor heterostructures offer a way to achieve large and designable nonlinearities with dynamic modulation of intersubband energies through the Stark effect. One promising approach for incorporating these nonlinearities into free space optics is a nonlinear polaritonic metasurface, which derives resonant coupling between intersubband nonlinearities and optical modes in nanocavities. Recent work has shown efficient frequency mixing at low pumping intensities, with the ability to electrically tune the phase, amplitude, and spectral peak of it. However, the spectral tunability of intersubband nonlinearities is constrained by the static spectral response of nanocavities. To overcome this limitation, we present nonlinear polaritonic metasurfaces for a broadband giant nonlinear response. This is achieved by combining a Stark tunable nonlinear response from a quantum-engineered semi-conductor heterostructure with arrays of three nanocavities with different resonant wavelengths. We experimentally demonstrate broadband second harmonic generation (SHG) and a shift in the peak SHG efficiency within the range of 8.9–10.6 μm by applying bias voltage. This work will provide a promising route for achieving broadband and electrically tunable nonlinearities in metasurfaces.

## 1 Introduction

Nonlinear metasurfaces offer a distinct advantage of relaxed phase-matching constraints and the ability to control the local phase, amplitude, and polarization state of the nonlinear response, in contrast to conventional nonlinear optics based on bulk nonlinear crystals [1], [2]. These unique properties in subwavelength-thin films present a promising avenue for the development of “nonlinear flat optics” as a practical alternative to conventional nonlinear optics. Nonlinear flat optics platforms based on plasmonic [3], [4], [5], [6] or dielectric metasurfaces [[Bibr j_nanoph-2023-0682_ref_007] [Bibr j_nanoph-2023-0682_ref_008] [Bibr j_nanoph-2023-0682_ref_009] [Bibr j_nanoph-2023-0682_ref_010] [Bibr j_nanoph-2023-0682_ref_011] [Bibr j_nanoph-2023-0682_ref_012] [Bibr j_nanoph-2023-0682_ref_013] [Bibr j_nanoph-2023-0682_ref_014] [Bibr j_nanoph-2023-0682_ref_015] [Bibr j_nanoph-2023-0682_ref_016] have recently gained considerable attention, with great potential for innovative applications such as nonlinear holography [7], [16], [17], [18], [19], optical encryption [20], [21], [22], nonlinear optical switching and modulation [23], [24], and new frequency generation based on nonlinear frequency mixing [1]. However, due to the intrinsically low nonlinear response of conventional nonlinear medium and short interaction length of the nonlinear metasurface, achieving efficient frequency conversion has been challenging.

To address this problem, a nonlinear polaritonic metasurface combining a plasmonic nanocavity and a multiple quantum well (MQW) layer has been developed [[Bibr j_nanoph-2023-0682_ref_025] [Bibr j_nanoph-2023-0682_ref_026] [Bibr j_nanoph-2023-0682_ref_027] [Bibr j_nanoph-2023-0682_ref_028] [Bibr j_nanoph-2023-0682_ref_029]. Intersubband transitions (ISTs) in an n-doped semiconductor heterostructure-based MQW can provide giant second-order ( $\chi_{\mathrm{zzz}}^{\left(2\right)}$ ) and third-order ( $\chi_{\mathrm{zzzz}}^{\left(3\right)}$ ) nonlinear response for the polarization along the surface normal direction (*z* direction here) [31], [32]. The nanocavity can produce significant near-field enhancement, substantially amplifying the intrinsic nonlinearities in the MQW and resulting in a giant nonlinear response to incoming light in free space. This marks a notable advancement in the development of highly efficient nonlinear metasurfaces. Recently, the nonlinear polaritonic metasurface, arising from the doubly resonant intersubband transitions, demonstrated a conversion efficiency of second harmonic generation (SHG) over 0.07 % at pump intensities in the tens of kW/cm^−2^ range [28]. Furthermore, going beyond the static limit of passive-type flat nonlinear optics, the nonlinear polaritonic metasurfaces using the quantum-confined Stark effect (QCSE) of intersubband nonlinearities, $\chi_{\mathrm{zzz}}^{\left(2\right)}\left(V\right)$ , has been reported. This approach offers a multifunctional capability, enabling dynamic manipulation of light and on-chip integration with other electronics [25]. By applying bias voltages to the MQW layer in the nonlinear polaritonic metasurface, intensity, and phase modulation of SHG were achieved.

However, nonlinear polaritonic metasurfaces, thus far, have shown a narrowband spectral response for efficient frequency conversion due to the characteristics of their two main elements: MQW and plasmonic nanocavity. Firstly, the giant intersubband nonlinearities can be obtained near the center wavenumber under doubly resonant ISTs configuration mainly used for SHG. The intersubband nonlinear susceptibility tensor element of the MQW is expressed using the Equation (1) [33], [34]:

(1) $$\begin{array}{ll} \chi_{\mathrm{zzz}}^{\left(2\right)}\left(\omega \to 2\omega \right) & \approx \frac{e^{3}\left(N_{e,1}-N_{e,2}\right)}{\varepsilon_{0}}\\ & \;\times \frac{z_{12}z_{23}z_{31}}{\begin{array}{c} \left(\hbar \omega_{31}-2\hbar \omega -i\hbar \gamma_{31}\right)\\ \left(\hbar \omega_{21}-\hbar \omega -i\hbar \gamma_{21}\right) \end{array}} \end{array}$$

where *ω* is the pump frequency, *e* is the electron charge, *N*_*e*1_ and *N*_*e*2_ are the averaged electron densities located at the first and second electron subbands, respectively. *ℏω*_*ij*_ = *E*_*ij*_, *ez*_*ij*_, and *ℏγ*_*ij*_ denote the IST energy, the transition dipole moment, and the linewidth, respectively, for the transition between electron subbands *i* and *j*. At the center wavenumber under doubly resonant condition, $\chi_{\mathrm{zzz}}^{\left(2\right)}$ shows its maximum value when 2*ℏω*_21_ = *ℏω*_31_. As we move away from the central wavenumber, the nonlinear response decreases sharply. Secondly, the plasmonic nanocavity induces the surface-normally polarized electric fields in the MQW layer through harmonic modes of plasmonic resonance. The effective second-order nonlinearity ( $\chi_{\mathrm{ijk}}^{\left(2\right)\text{eff}}$ ) out of the nonlinear polaritonic metasurface is expressed by the following Equation (2) [30].

(2) $$\chi_{\mathrm{ijk}}^{\left(2\right)\text{eff}}=\chi_{\mathrm{zzz}}^{\left(2\right)}\left[\int_{u_{\text{MQW}}}\xi_{z\left(i\right)}^{2\omega }\xi_{z\left(j\right)}^{\omega }\xi_{z\left(k\right)}^{\omega }du\right]/u_{\text{unit}}$$

where the letter, *i*, refers to the polarization state of output second harmonic (SH) electric field and the last two letters, *j*, *k*, refer to the polarization states of input fundamental frequency (FF) electric field. $\xi_{z\left(i\right)}^{\omega \;\;\text{or}\;\;2\omega }$ is the ratio of induced *z*-polarized *E* field in the MQW region to the incident *i*-polarized *E* field at FF *ω* or SH frequency 2*ω*, and *u*_MQW_, *u*_unit_ represent the volume of MQW in the unit cell before and after MQW etching, respectively. The term, $f_{\mathrm{ijk}}\equiv \left[\int_{u_{\text{MQW}}}\xi_{z\left(i\right)}^{2\omega }\xi_{z\left(j\right)}^{\omega }\xi_{z\left(k\right)}^{\omega }du\right]/\text{d}u_{\text{unit}}$ , represents the modal overlap factor of Ez field enhancement distributions induced within the MQW layer at FF and SH frequencies by plasmonic resonances, exhibiting significant values only in a narrow spectral region. The effective nonlinear susceptibility, $\chi_{\mathrm{ijk}}^{\left(2\right)\text{eff}}$ , is expressed as the product of the modal overlap factor, *f*_*ijk*_, and the intrinsic nonlinear response of the MQW, $\chi_{\mathrm{zzz}}^{\left(2\right)}$ . Due to the resonant characteristics of each, $\chi_{\mathrm{ijk}}^{\left(2\right)\text{eff}}$ also exhibits a narrow spectral response.

**Figure 1:**
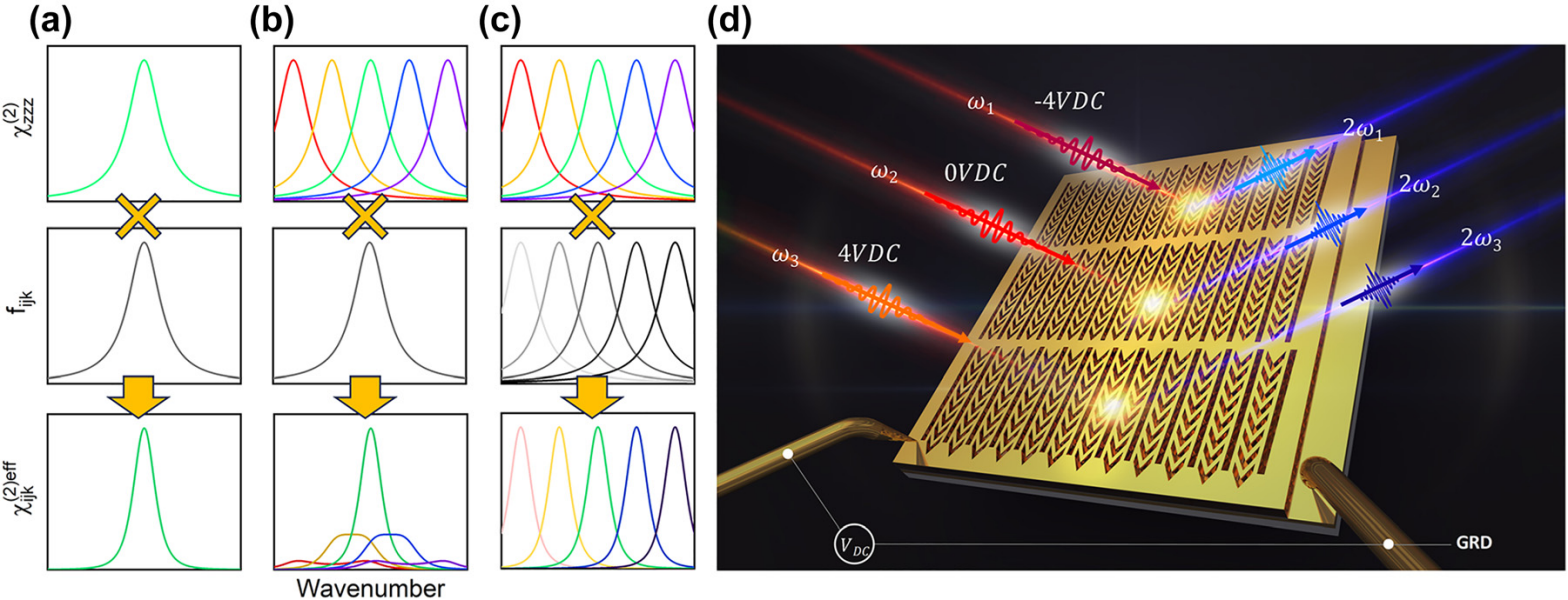
Concept of the electrically tunable nonlinear metasurface. (a–c) Flow scheme illustrating the electrically tunable nonlinear polaritonic metasurface for broadband SHG. $\chi_{\mathrm{ijk}}^{\left(2\right)\text{eff}}$ (bottom) can be expressed as the product of $\chi_{\mathrm{zzz}}^{\left(2\right)}$ (top) and *f*_*ijk*_ (center) for the case of (a) a passive metasurface, (b) an active metasurface with a single array of meta-atom, and (c) an active metasurface with arrays of meta-atoms. As voltages are applied to the MQW layer from negative to positive DC bias, the color of $\chi_{\mathrm{zzz}}^{\left(2\right)}$ spectrum changes from red (lower frequency) to purple (higher frequency). As the meta-atom structure is scaled upward, the brightness of the *f*_*ijk*_ spectrum increases. (d) The concept of the metasurface is composed of three arrays of meta-atom structures with an inserted MQW layer. Each array of meta-atom structures is optimized to have plasmonic resonance at the input pump frequencies of *ω*_1_, *ω*_2,_ and *ω*_3_, respectively.

Figure 1(a)–(c) show $\chi_{\mathrm{zzz}}^{\left(2\right)}$ (top), *f*_*ijk*_ (middle) and $\chi_{\mathrm{ijk}}^{\left(2\right)\text{eff}}$ (bottom) for the passive-type nonlinear polaritonic metasurface (Figure 1(a)), the active-type nonlinear polaritonic metasurface with only electrically tunable $\chi_{\mathrm{zzz}}^{\left(2\right)}$ (Figure 1(b)), and the nonlinear polaritonic metasurface with both electrically tunable $\chi_{\mathrm{zzz}}^{\left(2\right)}$ and arrays having different *f*_*ijk*_ proposed in this work (Figure 1(c)), respectively. In the case of passive-type nonlinear polaritonic metasurface as shown in Figure 1(a), due to the fixed $\chi_{\mathrm{zzz}}^{\left(2\right)}$ and *f*_*ijk*_, high conversion efficiency can be obtained only in a narrow wavelength range. Although previous work on electrically tunable nonlinear polaritonic metasurface reported a spectral shift of intersubband nonlinearities, $\chi_{\mathrm{zzz}}^{\left(2\right)}\left(V\right)$ , using the QCSE of IST energy as shown Figure 1(b), the measured spectral peak of nonlinear signal was still obtained in a narrow band. This is because the term of modal overlap integration restricts the total spectral tunability.

In this work, we experimentally demonstrated nonlinear polaritonic metasurfaces composed of arrays of meta-atom structures with slightly different dimensions to achieve a broadband nonlinear optical response. We achieved efficient frequency conversion and a broadband nonlinear response by applying different bias voltages to each meta-atom array. In numerical simulations, we achieved an effective second order nonlinear susceptibility of over 200 nm/V from 730 cm^−1^ to 1120 cm^−1^, with a more than threefold broader full-width half- maximum compared to a passive-type nonlinear polaritonic metasurface with a single meta-atom structure. We obtained over 0.025 % of SH power conversion efficiency in a broadband range of 924–1153 cm^−1^ out of two meta-atom arrays (M1 and M2). Significant spectral peak tuning of SHG in the mid-infrared (MIR) region was achieved for an input pump wavelength range from 945 to 1135 cm^−1^ for M1 and M2. This represents an approximately eightfold increase in SH power spectral peak shift compared to the previous nonlinear polaritonic metasurface (995–971 cm^−1^). Our results present a promising strategy for developing electrically tunable nonlinear polaritonic metasurface for efficient SHG in a broadband range, with potential implications for nonlinear flat optics and its applications.

## 2 Results

### 2.1 Numerical demonstration

The schematic image in Figure 1(d) represents the concept of the electrically tunable nonlinear metasurface for broadband SHG. The metasurface consists of arrays of meta-atom structures with different dimensions, incorporating a 400-nm-thick MQW layer located in a metallic nanocavity. Two metallic layers (top plasmonic nanoresonators, bottom ground plane), connected to electrodes, apply bias voltages to the MQW layer. The MQW layer is designed to have a giant second-order nonlinear response for SHG, and its maximum nonlinear response is spectrally tuned by the applied bias voltage, resulting in a broadband giant nonlinear response. By applying a bias voltage, the IST energies are tuned. Thus, the center wavenumber of the maximum SHG from metasurfaces can be tuned, and arrays of scaled meta-atoms targeting each wavenumber operate in sequence according to the bias voltage. For negative and positive DC bias voltages applied to the device, the maximum SHG occurs at higher and lower pump frequencies (*ω*_1_ < *ω*_2_ < *ω*_3_) from the targeted array of meta-atom structures, respectively. Therefore, the spectral peak of SHG is broadly tuned according to the bias voltage applied to the metasurface.

**Figure 2:**
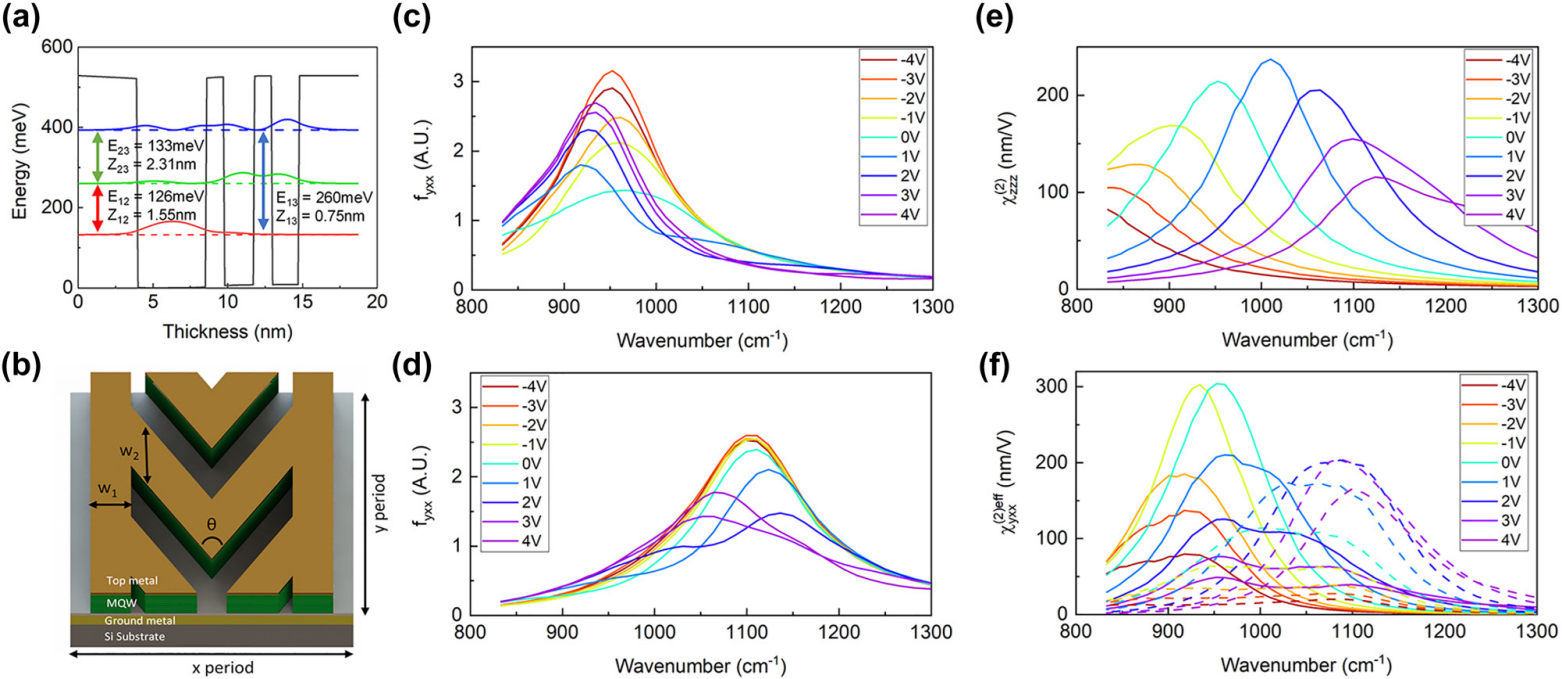
Meta-atom structure design and numerical simulations. (a) Conduction band diagram of the coupled three-quantum-well unit structure under zero bias voltage, where *E*_*ij*_ and *z*_*ij*_ indicate the transition energy and dipole element between electron states *i* and *j*, respectively. (b) Meta-atom structure designed for SHG. (c, d) Calculated modal overlap factor for different bias voltages as a function of the wavenumber for (c) M1 and (d) M2 structure, respectively. (e) Intrinsic second order susceptibility of the MQW and (f) effective second order susceptibility of M1 (solid line) and M2 (dotted line) for different bias voltages.

The In_0.53_Ga_0.47_As/**Al**_**0.48**_ **In**_**0.52**_ **As** heterostructure-based MQW unit structure (unbiased), shown in Figure 2(a), was designed by using a self-consistent Poisson–Schrodinger solver. The coupled three quantum well unit structure, with three quantized electron subbands, is designed for the electrical tuning of the giant second-order nonlinear response. The layer sequence of the quantum well unit structure is **4**/4.6/**1.2**/2/**1.2**/1.8/**4** (barrier sequence **Al**_**0.48**_ **In**_**0.52**_ **As** is noted in bold type, and 4 × 10^18^ cm^−2^ of n-type dopant is implanted in the first 4.6 nm thick In_0.53_Ga_0.47_As quantum well) in nanometers. The coupled three-quantum-well structure has a fascinating ability to manipulate the IST energies, inducing a large shift of the spectral peak of $\chi_{\mathrm{zzz}}^{\left(2\right)}\left(V\right)$ according to the bias voltage through the QCSE. Supplementary Figure S1(a) and (b) present the conduction band diagrams corresponding to applied bias voltages of +4 V and −4 V, respectively, over the 400 nm-thick MQW layer. The calculated IST parameters, permittivity, and second-order nonlinear response are presented in Supplementary Table S1 and Figure S2. The designed MQW was grown on the semi-insulating InP substrate by molecular beam epitaxy (MBE). The IST energies were measured through intersubband absorption measurements and are also presented in Supplementary Table S2. The measured values of *E*_21_ and *E*_31_ were found to be approximately 13 and 19 meV smaller than the theoretically calculated values.

The meta-atom structures for broadband nonlinear response of the nonlinear polaritonic metasurface were designed as shown in Figure 2(b). The V-shaped nanoantenna with two load lines at the lateral end is repetitively arranged in the *x* and *y* directions, where the neighboring unit cells are connected to the electrode in the *y* direction. The meta-atom structure induces the first-order plasmonic mode at the FF for *x*-polarization input and the first-order plasmonic mode at the SH frequency for *y*-polarization input. In this meta-atom structure, length (*L*) and the bending angle (*θ*) are the dominant factors for adjusting the plasmonic resonant wavelengths at the FF and SH frequency. The same meta-atom structure, optimized to have maximum $f_{\mathrm{yxx}}\left(V\right)$ at 1000 cm^−1^ in our previous study, was used as the reference dimension [25]. We scaled the dimensions along the *x*-axis of the meta-atom up and down from 85 % to 145 % with a 5 % scale interval. The dimensions along the *y*-axis and *θ* of the meta-atom are adjusted to achieve the maximum value of $f_{\mathrm{yxx}}\left(V\right)$ at each corresponding wavenumber. The field distributions and modal overlaps of 90 % and 115 % scaled meta-atom strucrues, monitored at 100 nm below the interface between the top metal resonator and the MQW layer, are shown in Supplementary Figure S3. These corresponding field distributions exhibit good modal overlap, with the two load line regions showing a high value of modal overlap integration, $f_{\mathrm{yxx}}\left(V\right)$ . In this configuration, the *yxx* polarization combination of effective second-order susceptibility ( $\chi_{\mathrm{yxx}}^{\left(2\right)\text{eff}}$ ) from the metasurface is obtained. Supplementary Figure S4 shows the $f_{\mathrm{yxx}}\left(V\right)$ and $\chi_{\mathrm{yxx}}^{\left(2\right)\text{eff}}$ spectra of each scaled meta-atom structure under targeted bias voltage. We simulated in the range of bias voltage from −4 V to +4 V. The peak value of $\chi_{\mathrm{zzz}}^{\left(2\right)}\left(V\right)$ decreases with increasing bias voltage, as the IST energies of *E*_21_ and *E*_31_ vary in different directions. The value of *f*_*yxx*_(*V*) increases with increasing bias voltage, as the intersubband absorption moves away from the peak of $\chi_{\mathrm{zzz}}^{\left(2\right)}\left(V\right)$ . Therefore, the peak value of $\chi_{\mathrm{yxx}}^{\left(2\right)\text{eff}}$ spectra, which is $\chi_{\mathrm{zzz}}^{\left(2\right)}\left(V\right)$ multiplied by *f*_*yxx*_ retains a value of over 200 nm/V in the broadband range. From this simulation result, we conclude that the nonlinear response from the meta-atom arrays is more than three times broader than that of a single nonlinear polaritonic metasurface, and it shows a higher effective susceptibility value in the broadband range compared to the electrically tunable nonlinear response of the MQW.

For experimental demonstration of the proposed concept, we selected two meta-atom structures, M1 and M2, to achieve an efficient frequency conversion in the operational range of a wavelength-tunable quantum cascade laser (tuning range: 909–1230 cm^−1^). The M1 meta-atom has dimensions scaled up by 15 % from the reference meta-atom structure, with *w*_1_ = 320 nm, *w*_2_ = 410 nm, *p*_*x*_ = 1670 nm, *p*_*y*_ = 1290 nm, *L* = 1380 nm, and *θ* = 61°. The M2 meta-atom has dimensions scaled down by 10 %, with *w*_1_ = 240 nm, *w*_2_ = 360 nm, *p*_*x*_ = 1300 nm, *p*_*y*_ = 1140 nm, *L* = 1080 nm, and *θ* = 55°. Based on their dimensions, the metasurfaces, M1 and M2, were optimized to have plasmonic resonance at pump wavenumbers of 928 cm^−1^ and 1100 cm^−1^, respectively.

The spectra of *f*_*yxx*_(*V*) for M1 and M2 are presented in Figure 2(c) and (d), respectively. The induced electric field by plasmonic resonance is influenced by intersubband absorption loss in the MQW layer, varying with bias voltage. Consequently, the spectrum of modal overlap integration changes in accordance with the bias voltage. The spectra of $\chi_{\mathrm{zzz}}^{\left(2\right)}\left(V\right)$ calculated using data in Supplementary Table S1 for bias voltages ranging from −4 V to 4 V are shown in Figure 2(e), and the spectra of $\chi_{\mathrm{yxx}}^{\left(2\right)\text{eff}}$ for M1 (solid line) and M2 (dashed line) are presented in Figure 2(f), respectively. In the case of M1, the spectral peak of $\chi_{\mathrm{yxx}}^{\left(2\right)\text{eff}}$ can be shifted from 917 to 964 cm^−1^ with a bias voltage range from −4 V to 4 V, with a maximum value of 328 nm/V at 940 cm^−1^ and a bias voltage of −0.8 V. For the M2 meta-atom structure, the spectral peak of $\chi_{\mathrm{yxx}}^{\left(2\right)\text{eff}}$ can be shifted from 944 to 1123 cm^−1^ by varying the bias voltage from −4 V to 4 V, with a maximum value of 214 nm/V at 1087 cm^−1^ and a bias voltage of 3.2 V. These metasurfaces provide an efficient and broadband nonlinear response of over 150 nm/V within the wavenumber range from 877 to 1141 cm^−1^, allowing for electrical control of the nonlinear response over a wide range.

### 2.2 Experimental demonstration

**Figure 3:**
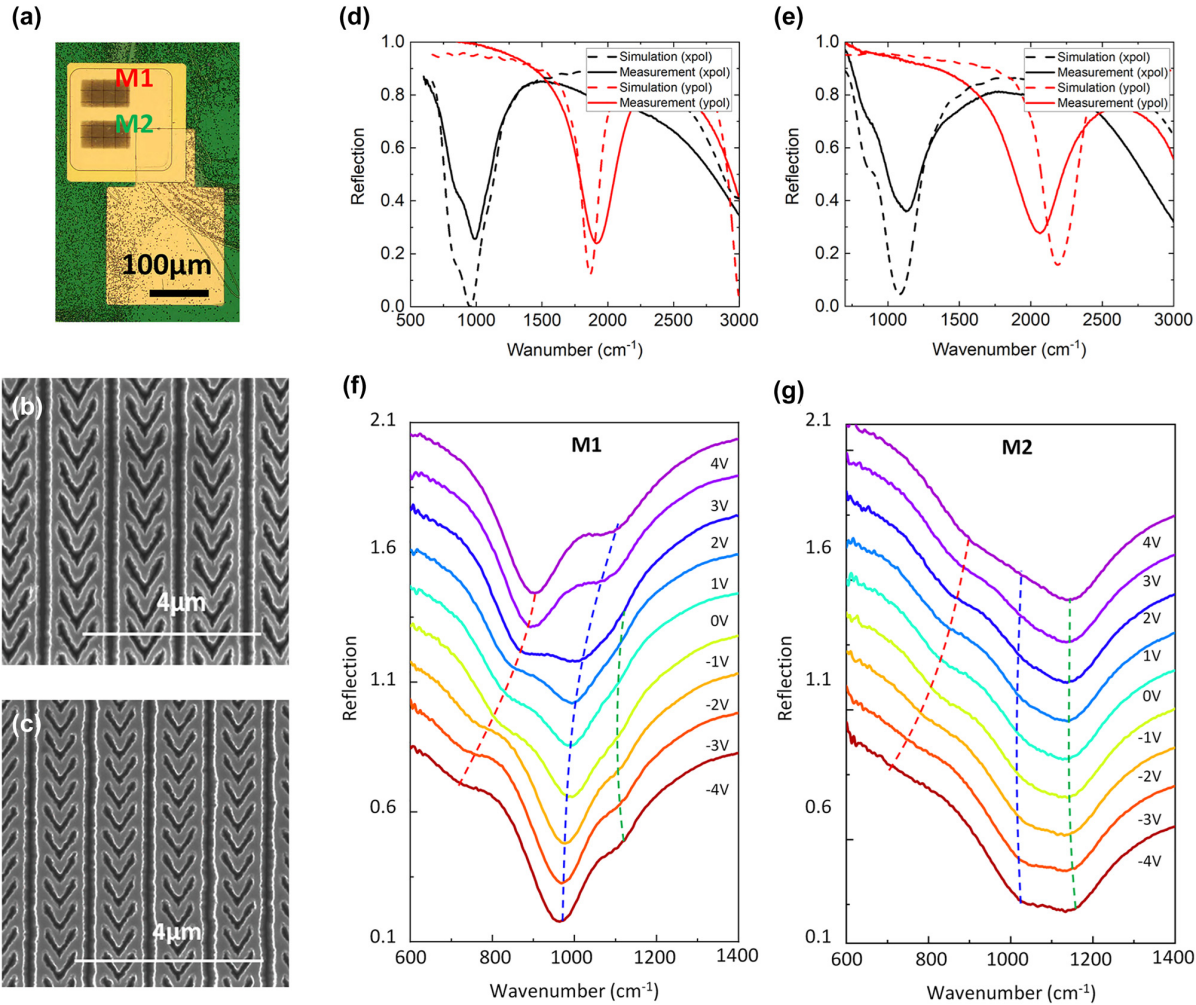
Linear characterization of the metasurface. (a) Optical microscope image and (b, c) scanning electron microscopy images of the fabricated metasurface (b) M1 and (c) M2. (d, e) Simulated (dashed curve) and measured (solid curve) linear reflection spectra of the metasurface (d) M1 and (e) M2 for *x*- (black) and *y*-polarized (red) light at normal incidence. (f, g) Reflection spectra of the arrays of the meta-atom structures, (f) M1 and (g) M2 optimized to have plasmonic resonance at 10.6 μm and 9.5 μm, respectively, under *x*-polarized incident light and a DC bias voltage ranging from −4 V to +4 V with 1 V step. For better display of the data, the reflection spectra at different bias voltages are offset from each other vertically by 0.15. The three dashed curves trace the positions of the three polaritonic peaks induced by the coupling of the plasmonic resonance, 1–2 level IST, and 2–3 level IST as the DC bias voltage changes.

To experimentally demonstrate the performance of our metasurface, we fabricated two 100 μm × 50 μm two-dimensional arrays (M1, M2) in a single device. Figure 3(a) shows an optical microscopy image of the fabricated metasurface. The M1 and M2 meta-atom arrays are patterned using electron-beam lithography and a two-step reactive-ion etching process. On the right, the lower side of the metasurface has a T-shaped contact electrode patterned using photolithography and a lift-off process. The metasurface is mesa-etched into a 250 μm × 250 μm square shape to prevent current leakage and is passivated by a 400 nm thick Si_3_N_4_ layer (shown in green region in Figure 3(a)) with a 200 μm × 200 μm pattern opening. The T-shaped contact pad is connected to the top metal layer, and the 400 nm thick Si_3_N_4_ layer isolates the contact pad from the bottom metal. To mitigate the effects of sample heating, we mounted the fabricated sample on a Cu plate heat sink and applied bias voltage using a 50 μm diameter probe tip. The scanning electron microscopy images of the fabricated metasurfaces (M1 and M2) are shown in Figure 3(b) and (c), respectively.

Figure 3(d) and (e) show simulated (dashed line) and measured (solid line) linear reflection spectra of the M1 and M2 metasurface for *x*- (black) and *y*-polarized (red) IR light at normal incidence, respectively. For incident light with *x*-polarization, we observe slight differences between the designed wavenumber of the modal overlap peak and the measured wavenumber of the reflection peak due to the strong resonant coupling of plasmonic cavity modes and ISTs at 925 and 1030 cm^−1^, respectively. The interaction between electrically tunable ISTs and the fixed resonance of the plasmonic nanoantenna modulates the peaks resulting from polaritonic splitting in the metasurface. To verify the tuning of ISTs and the corresponding polaritonic peak tuning, linear reflection spectra for the two metasurfaces under the *x*-polarized incident light were measured by applying DC bias voltages ranging from −4 V to +4 V with a 1 V step, as shown in Figure 3(f) and (g). It is noteworthy that applying a voltage higher than ±4 V results in several hundred mA flowing through the device, leading to issues such as sample burning or short-circuiting. The blue and red dashed curves in Figure 3(f) demonstrate that the polaritonic peak splitting, caused by the strong coupling between the plasmonic resonance and the *E*_21_ IST, was tuned by adjusting the bias voltage from +4 V to −4 V based on the red-shifted *E*_21_ IST wavelength. When the negative bias voltage was increased, an additional peak splitting (blue and green dashed curves) resulting from the coupling with the *E*_32_ IST was also observed. In the case of the M2 metasurface, the coupling with the *E*_12_ IST was weak because the plasmonic resonance was apart from the *E*_12_ IST. However, polaritonic splitting due to coupling with the *E*_23_ IST near a wavelength of 1111 cm^−1^ (9 μm) was observed as the negative bias increased. These results confirmed that the ISTs tuned by the bias voltage agreed well with the calculation results. Due to the polaritonic coupling effect, which can be controlled via the bias voltage, it is possible to generate broadband efficient SHG in the 8–11 μm pump wavelength range using the two metasurfaces. The linear spectral tuning of the metasurfaces was well matched with simulated reflection spectra (see Supplementary Figure S5). The linear reflection spectra near the SH frequency for the *y*-polarized incident light and different bias voltages were simulated and measured, as shown in Supplementary Figures S6 and 7, respectively. The resonant absorption peak near the SH frequency exhibits no spectral tuning regardless of the applied bias voltage.

**Figure 4:**
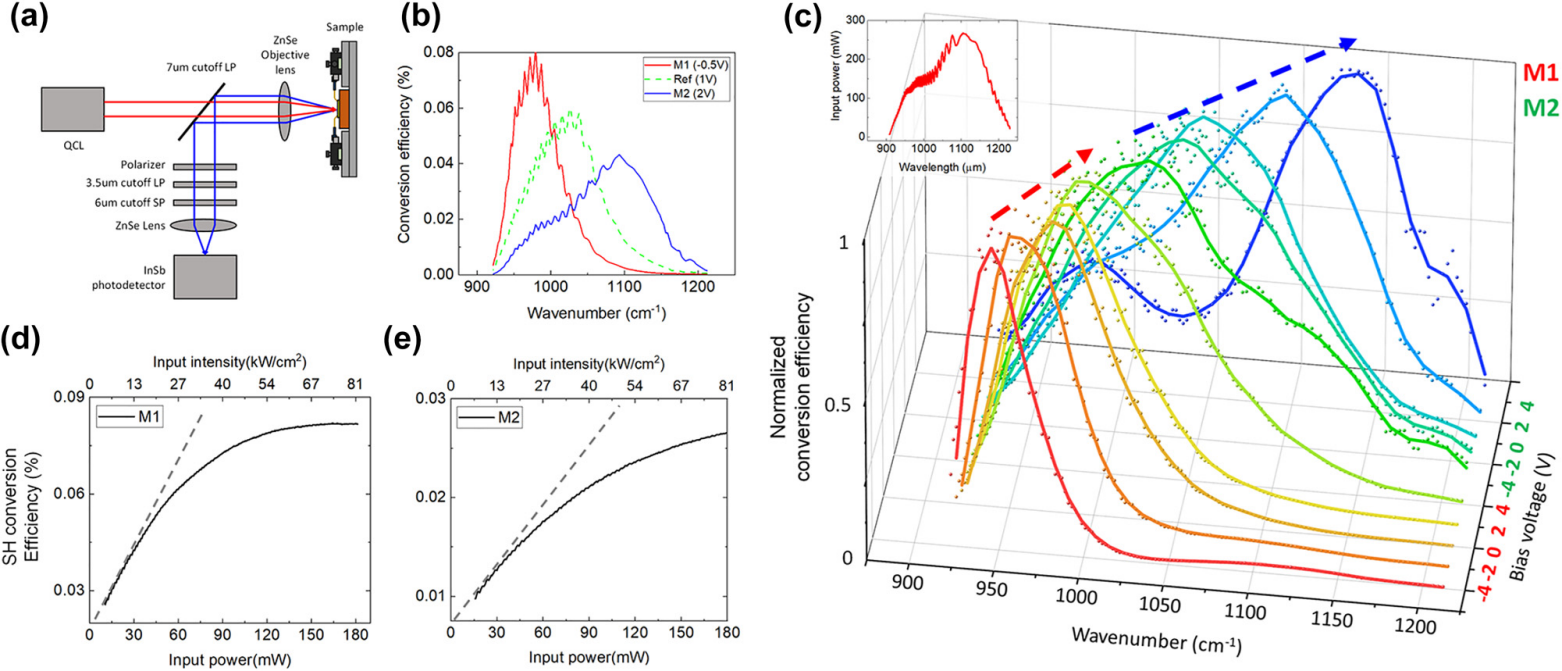
Nonlinear characterization of the electrically tunable metasurface. (a) The optical setup for the measurement of SHG response of the nonlinear polaritonic metasurface under a DC bias voltage. (b) Measured SH power conversion efficiency spectra under bias voltage for maximum of SHG value of each metasurfaces, M1: red solid line, reference structure: green dotted line and M2: blue solid line. (c) 3D plot of the measured SHG conversion efficiency spectra normalized to their maximum value as a function of the input pump wavenumber for different DC bias voltages from −4 V to +4 V with 2 V step. (d, e) measured SH power conversion efficiency as a function of the input pump power for the metasurfaces (d) M1 and (e) M2 at pump wavelength of 975 and 1095 cm^−1^, respectively.

The optical setup to measure the SHG signal from the nonlinear polaritonic metasurface under a DC bias voltage is illustrated in Figure 4(a). A wavelength-tunable quantum cascade laser (QCL) was used as an input pump. The linearly polarized input beam at the FF (red line) from the QCL was focused onto the metasurface via the long-pass filter (LP) and the ZnSe objective lens. The SH signal (blue line) generated from the device was collected by the ZnSe objective lens and measured using the InSb photodetector. The unwanted harmonic signals from the QCL were filtered by the LP with a cutoff wavelength of 7 μm. The *x*-polarized QCL input beam reflected from the device was filtered by the LP with cutoff wavelength of 7 μm, a short-pass filter (SP) with cutoff wavelength of 7 μm, and a linear polarizer. The higher-order nonlinear beam generated by the device was also filtered by another LP with a cutoff wavelength of 3.5 μm and the linear polarizer. Figure 4(b) shows the measured SHG conversion efficiency spectra for the M1, M2, and the reference meta-atom structure (dashed line) at voltages of −0.5 V, 2 V, and 1 V, respectively, obtaining the maximum SHG efficiency for each structure at these respective voltages. The peak SHG conversion efficiency values for the M1 and M2 metasurfaces corresponding to the bias voltage are provided in Supplementary Figure S8. In the case of M1, the spectral peak of $\chi_{\mathrm{yxx}}^{\left(2\right)\text{eff}}$ can be shifted from 945 to 990 cm^−1^ by varying the bias voltage from −4 V to 4 V, reaching a maximum value of 0.092 % at 975 cm^−1^ with a bias voltage of −0.5 V. For the M2 structure, the spectral peak of $\chi_{\mathrm{yxx}}^{\left(2\right)\text{eff}}$ can be shifted from 1021 to 1134 cm^−1^ within a bias voltage range of −4 V–4 V, reaching a maximum value of 0.041 % at 1095 cm^−1^ with a bias voltage of 2 V. Two metasurfaces, M1 and M2, exhibit efficient and broadband nonlinear responses, achieving over 0.025 % of SH power conversion efficiency in the broad range of 924–1153 cm^−1^.

Figure 4(c) represents SHG conversion efficiency spectrum normalized to the maximum value of itself. Experimentally, broadband SHG spectral peak tuning was achieved for the 945–1134 cm^−1^ input pump wavelength range (10.6–8.8 μm in wavelength). The inset in the top left of Figure 4(c) shows the power spectrum of the QCL. The measured SHG power conversion efficiencies as a function of input peak power and input peak intensity for the two metasurfaces at their optimal operating pump wavelengths (975, 1095 cm^−1^ for M1, M2, respectively) at 0 V are shown in Figure 4(d) and (e). Both metasurfaces exhibit a SHG power conversion efficiency exceeding 0.025 % with an input pump peak power of 180 mW and a peak intensity of 81 kW cm^−2^. For the M1 metasurface at the equivalent pump power, an SHG conversion efficiency of 0.082 % corresponding to an SHG peak power of 85 μW was achieved. At a pump intensity above 25 kW cm^−2^, the slope of the SHG conversion efficiency curve decreases owing to the SHG intensity saturation effect. From the slope of this graph, the $\chi_{\mathrm{yxx}}^{\left(2\right)\text{eff}}$ of two metasurfaces can be extracted. The nonlinear conversion factor $\eta =P_{\text{SH}}/{\left(P_{\text{pump}}\right)}^{2}$ for the two metasurfaces at low (high) pump intensity was 9.0 (2.8) mW W^−2^ for the M1 metasurface and 2.8 (0.6) mW W^−2^ for the M2 metasurface, and the corresponding $\chi_{\mathrm{yxx}}^{\left(2\right)\text{eff}}$ for the two metasurfaces at low (high) pump intensity was 304 nm/V (94) mW W^−2^ for the M1 metasurface and 155 nm/V (33) mW W^−2^ for the M2 metasurface.

## 3 Conclusions

We demonstrated that a nonlinear polaritonic metasurface, composed of arrays of meta-atom structures, has the ability to achieve efficient nonlinear conversion in the broadband range. We experimentally demonstrated electrically tunable nonlinear metasurfaces for broadband efficient SHG where the two meta-atom arrays (M1 and M2) in a single device operate sequentially according to the applied voltage. By deploying more arrays of meta-atom structures, one can attain a more precise and broader nonlinear response in MIR range. Due to the operational range of the QCL and unexpected CO_2_ atmospheric absorption (2*ω* > 2242 cm^−1^) at SH frequency, we demonstrated the concept of a nonlinear polaritonic metasurface using two arrays of M1 and M2. The proposed approach is not limited to SHG and can be applied to other nonlinear optical processes, such as sum- and difference-frequency generation and third-harmonic generation. It is also possible to extend the method to near-IR region by using materials that can induce larger conduction band offsets. The nonlinear metasurfaces demonstrated here may provide a new device platform that significantly expands the design space of nonlinear flat optics, thanks to their electrically tunable broadband giant nonlinear response. Additionally, they offer a pathway to implement innovative meta-devices, including electrically tunable broadband nonlinear light sources, active nonlinear beam manipulation, electrically tunable nonlinear holography, and ultrafast nonlinear signal switching and modulation.

## 4 Experimental section

### 4.1 Device fabrication

To transfer the MQW layer to the bottom metallic layer deposited on the Si substrate, a sequence of metallic layers, including 20 nm of Cr, 50 nm of Pt, and 100 nm of Au, are deposited on both surfaces of the MQW layer and the Si substrate. A pressure of 1.2 kN/cm^−2^ and a heat of 240 °C are applied for Au–Au vacuum metal welding. The InP substrate is selectively etched using HCl:H_2_O solution, followed by a selective wet etching process removing 300 nm of InGaAs layer using H_3_PO_4_:H_2_O_2_:H_2_O solution, and 100 nm of InP layer using HCL:H_2_O solution. For the top metallic layer, 5 nm of Cr and 50 nm of Au are evaporated onto the exposed MQW surface. A 400 nm of Si_3_N_4_ layer is deposited by plasma-enhanced chemical vapor deposition on the top metallic layer to serve as a mask for selective dry etching. The meta-atom patterns are then written on the sample using electron beam lithography with ARP-6200.09 resist. The Si_3_N_4_ layer is dry-etched following the pattern of the ARP-6200.09 resist mask using an inductive coupled plasma-type reactive ion etching (RIE) tool with CHF_3_, CH_4_ and N_2_ gases. The metal-MQW-metal meta-atom structure is subsequently dry-etched using Cl_2_, Ar and N_2_ gases. The metasurface device is mesa-etched into a 250 μm × 250 μm square shape to prevent current leakage. This is achievced by creating a photo mask pattern through photolithography and subsequent dry etching using RIE. The device is then passivated with a 400 nm-thick Si_3_N_4_ layer, which includes a 200 μm × 200 μm opening. A T-shaped contact electrode, composed of 20 nm of Cr, 300 nm of Cu, 10 nm of Cr, and 50 nm of Au, is patterned using photolithography and a lift-off process. This electrode is connected to the top metal layer of the metasurface. The fabricated metasurfaces device is attached to a thick Cu plate using silver paste, establishing an electrical connection between the bottom metallic layer and the Cu plate. The Cu plate serves as both a thermal sink and a ground electrode.

### 4.2 Optical characterization

The mid-infrared (from 600 to 5000 cm^−1^) reflection spectra were measured using a FTIR spectrometer equipped with an IR microscope (Bruker, vertex 70 and hyperion 1000). To apply a stable bias voltage to sample, a custom probe stage with a source meter (Keithley, SMU 2450) was employed. For nonlinear optical measurements, a broadly wavelength-tunable QCL operating in pulse mode (Daylight Solutions Inc., MIRCAT system, tuning range: 909–1230 cm^−1^, peak power: 400 mW, repetition rate: 100 kHz, duty cycle: 10 %) and a calibrated InSb photodetector (Electro Optical System, Inc.,) were used. The linearly polarized input beam from the QCL passes through the LP filter (cut-off wavelength: 7 μm) and focuses on the metasurface by the ZnSe objective lens (numerical aperture: 0.45). The generated SH signal was directed to the detector via the LP filter, the linear polarizer, the ZnSe lens, the SP filter (cut-off wavelength: 6 μm), and LP filter (cut-off wavelength: 3.5 μm). The focal spot diameter at the sample position was 2*w* = 24 μm, confirmed by the knife-edge measurement. A Gaussian power profile was assumed for both the FF input pump beam ( $P_{\text{FF}}=I_{\text{FF}}e^{-2r^{2}/w^{2}}$ ) and the SH beam ( $P_{\text{SH}}=I_{\text{SH}}e^{-4r^{2}/w^{2}}$ ). The average pump power was measured by a thermal power meter (Thorlabs, S302C). The DC bias voltage was applied using a source meter (Keithley, SMU 2450).

## Supplementary Material

Supplementary Material Details
