# Supplementary material for: Broadband giant nonlinear response using electrically tunable polaritonic metasurfaces
Source: Nanophotonics. 2024 Jan 9;13(7):1131–9. doi: 10.1515/nanoph-2023-0682 (PMC11502031; doi:10.1515/nanoph-2023-0682)
Supplement: Supplementary file 1 — Supplementary Material Details [file j_nanoph-2023-0682_suppl_001.docx]

Supporting Information for

**Broadband giant nonlinear response using electrically tunable polaritonic metasurfaces**

Jaeyeon Yu^1^, Seongjin Park^1^, Inyong Hwang^1^, Gerhard Boehm^2^, Mikhail A. Belkin^2^, and Jongwon Lee^1*^

^1^Department of Electrical Engineering, Ulsan National Institute of Science and Technology (UNIST), Ulsan 44919, Republic of Korea

*^2^Walter Schottky Institute, Technical University of Munich, Am Coulombwall 4, Garching 85748, Germany*

**Figure S1.** The conduction-band diagram of a single period of the MQW heterostructure used in this work for applied bias voltages of (a) +4 (100 kV/cm) and (b) −4V (−100 kV/cm), respectively, over a 400-nm-thick MQW layer. The applied bias voltage across the MQW layer tilts the conduction band edge, corresponding to the electric field. The IST energy, *E_ij_*, and dipole matrix element, *Z_ij_*, (between electron subbands *i* and *j*) can then be tuned by the bias voltage applied to the MQW layer through the quantum-confined Stark effect.

**Table S1.** Calculated IST parameters (IST energy, *E_ij_*, and dipole matrix element, *Z_ij_*) for the first three electron subbands for different bias voltages obtained using a self-consistent Poisson Schrödinger solver. We assumed that working temperature is 300K.

**Table S2.** Extracted IST parameters (IST energy, *E_ij_*, effective carrier density, *N_e_*_,1_, linewidth, *2ħϒ_ij_*) from intersubband absorption measurement for the first three electron subbands at 0V.

**Figure S2.** (a) IST energy of E_21_ (black) and E_31_ (blue) as a function of bias voltage. (b) Calculated nonlinear susceptibility,$\chi_{MQWs,zzz}^{(2)}$, of the MQW structure as a function of bias voltage. (c-e) Calculated dielectric constant of the MQW structure for the surface parallel direction () and surface normal direction ( or z-direction here) using the IST energies and dipole matrix element among the first three electron states at bias voltages ranging from -4V to +4V.


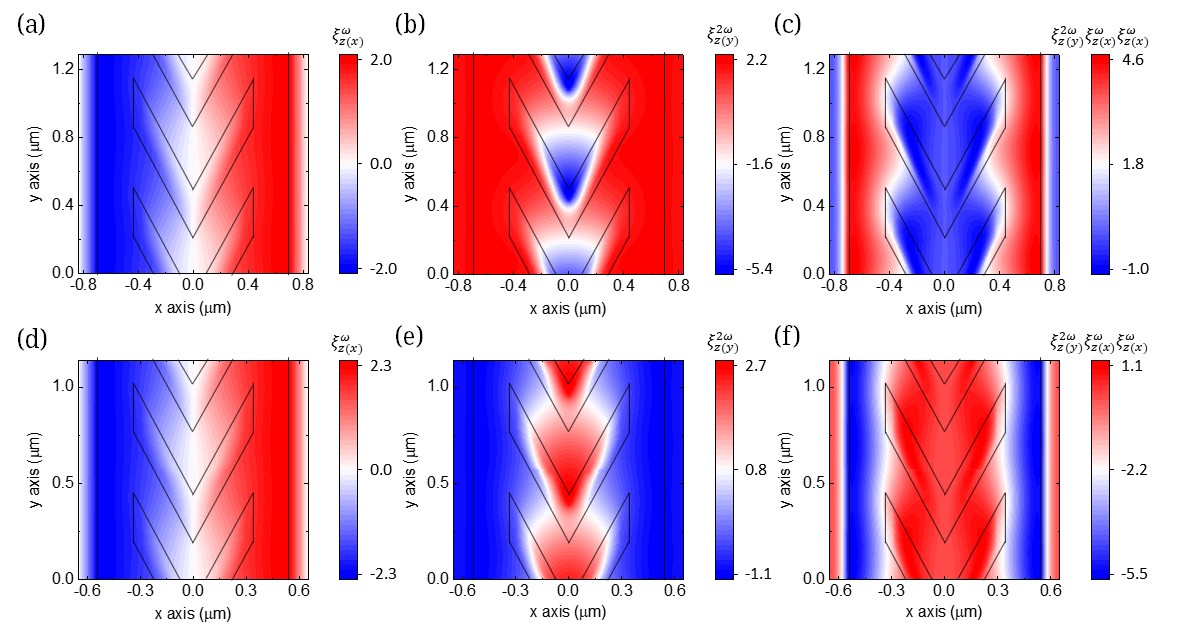


**Figure S3.** (a, d) *z*-polarized near-field enhancement distributions under the *x*-polarized input E-field at frequency *ω* ($\xi_{z(x)}^{\omega}$) and (b, e) *z*-polarized near-field enhancement distributions under the *y*-polarized input E-field at frequency *2ω* ($\xi_{z(y)}^{2\omega}$) monitored at 100 nm below from the top surface of the MQW layer. Black lines in the figures are the edge of the nanoresonator pattern. (c, f) Modal overlap distributions ($\xi_{z(y)}^{2\omega}\xi_{z(x)}^{\omega}\xi_{z(x)}^{\omega}$) used to compute $\chi_{ijk}^{(2)eff}$ in equation (2). The highest effective nonlinear susceptibility of the metasurface is produced for the *yxx* polarization combination, where the first letter refers to SH polarization and the last two letters refer to FF input pump polarization.

**Figure S4.** At first, to minimize IST coupling with plasmonic resonance and confirm the center wavenumber of plasmonic resonance, we calculated the modal overlap factor, (a) $f_{yxx}$, without the intersubband absorption of the MQW layer. Without the intersubband absorption in the MQW, it is assumed that the permittivity of the MQW is isotropic with the permittivity in the parallel direction. The brightness was adjusted and displayed in order of meta-atom size from smallest to largest, and color dots from purple to red are located on the peak in order of meta-atom size from smallest to largest. Due to the fixed thickness and size limitation imposed by the etching profile, linewidth of modal overlap spectra at each target wavenumber becomes broader with its wavenumber. (b) Spectra of intrinsic second order susceptibility of MQW as a function of bias voltage. The bias voltage was set to have the same peak position as the modal overlap integration peak of each meta-atom. (c) Modal overlap integration $f_{yxx}(V)$ with intersubband absorption. The dimension of the meta-atom structure is the same as the meta-atom structure in (a). Optical loss from intersubband absorption reduces the induced electric field in MQW layer. The doted point in (c) shows that the value $f_{yxx}\left( V \right)$ at the whavenumber of the peak of $\chi_{zzz}^{(2)}(V)$ in (b) and peak of $f_{yxx}$ in (a). There is a tradeoff between the value of $f_{yxx}(V)$ and the value of $\chi_{zzz}^{(2)}(V)$ near where intersubband absorption by E_21_ coincides with the peak position of $\chi_{zzz}^{(2)}(V)$ at the wavenumber of 1040 cm^-1^. (d) Calculated spectra of $\chi_{yxx}^{\left( 2 \right)eff}(V)$ of each meta-atom with corresponding voltage.

**Figure S5.** (a, b) Simulated reflection spectra of arrays of the meta-atom structures (a) M1 and (b) M2 that are optimized to have plasmonic resonance at 10.5 μm and 9.8 μm, respectively, under the *x*-polarized incident light and the DC bias voltage ranging from -6V to +2V with 1V step. For better display of the data, the reflection spectra at different bias voltages are offset from each other vertically by 0.3. In this simulation, IST energies calculated from the Poisson-Schrodinger solver were used.

**Figure S6.** (a, b) Simulated reflection spectra of arrays of the meta-atom structures (a) M1 and (b) M2 near SH wavelength under the *y*-polarized incident light and the DC bias voltage ranging from -6V to +2V with 1V step. For better display of the data, the reflection spectra at different bias voltages are offset from each other vertically by 0.3. In this simulation, IST energies calculated from the Poisson-Schrodinger solver were used.

 **Figure S7.** (a, b) Measured reflection spectra of arrays of the meta-atom structures (a) M1 and (b) M2 near SH wavelength under the *y*-polarized incident light and the DC bias voltage ranging from -4V to +4V with 1V step. For better display of the data, the reflection spectra at different bias voltages are offset from each other vertically by 0.15.

**Figure S8.** (a, b) Experimentally measured SHG peak conversion efficiency for the meta-atom structures, (a) M1 and (b) M2 as a function of the applied DC bias voltage ranging from -4V to +4V with 1V step. For the meta-atom structure of M1, the spectral peak of $\chi_{ijk}^{(2)eff}$can be shifted from 945 to 990 cm^-1^, and for M2, the spectral peak of $\chi_{ijk}^{(2)eff}$ can be shifted from 1021 to 1134 cm^-1^ with a bias voltage range from -4 V to 4 V, respectively.
